# The Use of Natural Language Processing to Assess Social Support in Patients With Advanced Cancer

**DOI:** 10.1093/oncolo/oyac238

**Published:** 2022-11-25

**Authors:** Sunil Bhatt, P Connor Johnson, Netana H Markovitz, Tamryn Gray, Ryan D Nipp, Nneka Ufere, Julia Rice, Matthew J Reynolds, Mitchell W Lavoie, Madison A Clay, Charlotta Lindvall, Areej El-Jawahri

**Affiliations:** Department of Medicine, Division of Hematology & Oncology, Massachusetts General Hospital, Boston, MA, USA; Department of Medicine, Division of Hematology & Oncology, Massachusetts General Hospital, Boston, MA, USA; Harvard Medical School, Boston, MA, USA; Department of Medicine, Division of Hematology & Oncology, Massachusetts General Hospital, Boston, MA, USA; Harvard Medical School, Boston, MA, USA; Department of Psychosocial Oncology and Palliative Care, Dana-Farber Cancer Institute, Boston, MA, USA; Department of Medicine, Division of Hematology & Oncology, Massachusetts General Hospital, Boston, MA, USA; Harvard Medical School, Boston, MA, USA; Harvard Medical School, Boston, MA, USA; Division of Gastroenterology, Department of Medicine, Massachusetts General Hospital, Brigham and Women’s Hospital, Boston, MA, USA; Department of Psychiatry, Massachusetts General Hospital, Boston, MA, USA; Department of Psychiatry, Massachusetts General Hospital, Boston, MA, USA; Department of Psychiatry, Massachusetts General Hospital, Boston, MA, USA; Department of Psychiatry, Massachusetts General Hospital, Boston, MA, USA; Harvard Medical School, Boston, MA, USA; Department of Psychosocial Oncology and Palliative Care, Dana-Farber Cancer Institute, Boston, MA, USA; Department of Medicine, Division of Hematology & Oncology, Massachusetts General Hospital, Boston, MA, USA; Harvard Medical School, Boston, MA, USA

**Keywords:** natural language processing, social support, hospitalization, survival analysis

## Abstract

**Background:**

Data examining associations among social support, survival, and healthcare utilization are lacking in patients with advanced cancer.

**Methods:**

We conducted a cross-sectional secondary analysis using data from a prospective longitudinal cohort study of 966 hospitalized patients with advanced cancer at Massachusetts General Hospital from 2014 through 2017. We used NLP to identify extent of patients’ social support (limited versus adequate as defined by NLP-aided review of the Electronic Health Record (EHR)). Two independent coders achieved a Kappa of 0.90 (95% CI: 0.84-1.00) using NLP. Using multivariable regression models, we examined associations of social support with: 1) OS; 2) death or readmission within 90 days of hospital discharge; 3) time to readmission within 90 days; and 4) hospital length of stay (LOS).

**Results:**

Patients’ median age was 65 (range: 21-92) years, and a plurality had gastrointestinal (GI) cancer (34.3%) followed by lung cancer (19.5%). 6.2% (60/966) of patients had limited social support. In multivariable analyses, limited social support was not significantly associated with OS (HR = 1.13, *P* = 0.390), death or readmission (OR = 1.18, *P* = 0.578), time to readmission (HR = 0.92, *P* = 0.698), or LOS (β = −0.22, *P* = 0.726). We identified a potential interaction suggesting cancer type (GI cancer versus other) may be an effect modifier of the relationship between social support and OS (interaction term *P* = 0.053). In separate unadjusted analyses, limited social support was associated with lower OS (HR = 2.10, *P* = 0.008) in patients with GI cancer but not other cancer types (HR = 1.00, *P* = 0.991).

**Conclusion:**

We used NLP to assess the extent of social support in patients with advanced cancer. We did not identify significant associations of social support with OS or healthcare utilization but found cancer type may be an effect modifier of the relationship between social support and OS. These findings underscore the potential utility of NLP for evaluating social support in patients with advanced cancer.

## Teaser

This study used natural language processing (NLP) to depict the extent of social support for a large cohort of hospitalized patients with advanced cancer. Associations were then examined, focusing on the extent of patients’ social support with their survival and healthcare utilization.

**Implications for Practice**

Natural language processing was used to assess the extent of social support in patients with advanced cancer. The authors found no significant associations of limited social support with survival or healthcare utilization; however, differential associations among cancer types were detected, including an association of limited social support with survival in those with gastrointestinal cancer. These findings support the use of natural language processing to successfully assess social support in patients with cancer. Future work is needed to further explore the integration of natural language processing into the electronic health record to assess complex health constructs, and larger scale studies in specific cancer types should further examine the impact of social support on outcomes.

## Introduction

Social support is a complex construct defined as the network of relationships that allow a person to cope with life stressors.^1^ Multiple studies have demonstrated patients’ self-report of social support correlates with all-cause mortality.^2-4^ In adults with aggressive hematologic malignancies, data suggest that limited social support is associated with worse overall survival (OS) and greater healthcare utilization.^5^ Patients with advanced cancer also face numerous stressors including high symptom burden, intensive treatment regimens, and frequent healthcare use.^6^ Improved social support may act as a protective factor enabling patients’ to better contend with these stressors. However, data are lacking to examine associations among patients’ social support, OS, and healthcare utilization in this patient population.^7^

Numerous and multifaceted limitations have historically made effectively evaluating social support in oncology difficult, including challenges in accurately assessing the amount of social support available to patients, flaws in self-reporting of social support (including ceiling effects),^8^ small sample sizes and missing data in patient-reported outcomes studies.^9,10^ Collectively, these limitations have deterred robust assessments of how patients’ social support influences their clinical outcomes.^7,11^ Prior studies have typically used marital status as a proxy for social support.^12^ However, this proxy does not fully encapsulate other important sources, such as assistance from family, friends, peers, and others in the community. Additionally, evaluating data on social networks often relies on unstructured text in the electronic health record (EHR), which makes codifying and extracting this data difficult.^13^ Thus, novel tools are needed to improve identification of information regarding patients’ social support.

Natural language processing (NLP) represents a promising method to overcome historical limitations for evaluating patients’ social support. Specifically, NLP allows for rapid extraction and analysis of relevant information contained in large volumes of text. NLP can detect prespecified indicators in the EHR,^14^ and thus has the potential to assess the extent of social support utilizing EHR. Recent studies have shown that NLP can help capture information about patient functional status and end-of-life quality indicators.^14-16^ Additionally, prior work has demonstrated the feasibility of using NLP to assess the extent of social support in patients with aggressive hematologic malignancies, further highlighting its potential use in oncology.^5^

In the present work, we used NLP to depict the extent of social support for a large cohort of hospitalized patients with advanced cancer. We also examined associations among the extent of patients’ social support with their survival and healthcare utilization in this population. We hypothesized that limited social support assessed by NLP would be associated with decreased survival and higher rates of healthcare utilization in patients with advanced cancer.

## Methods

### Study Procedures

This study was approved by the Dana-Farber/Harvard Cancer Center institutional review board. This is a cross-sectional secondary data analysis of a parent study that enrolled adult patients with cancer who were hospitalized for an unplanned hospital admission at Massachusetts General Hospital (MGH) in a longitudinal cohort study from 9/2014 to 4/2017.^17-19^ We identified and recruited consecutive patients with an unplanned hospital admission (index hospitalization) during the study period by screening the daily inpatient oncology census. Each participant contributed one unique hospitalization, with repeated hospitalizations excluded from analysis. A research assistant obtained written, informed consent from eligible patients within 5 days of the hospitalization. In this cross-sectional analysis, we focused on individuals with advanced solid tumors, which included 966 patients.

### Participants

Participants in this secondary analysis were eligible if they were adults (age ≥18), diagnosed with advanced cancer, and admitted to MGH. Advanced cancer was defined as not being treated with curative intent for a solid tumor malignancy based on the systemic therapy order entry treatment intent designation (palliative vs. curative) or based on documentation in the oncology clinic notes for those not receiving systemic therapy. Participants also had to be able to read and speak English well enough to independently complete study questionnaires. We excluded patients admitted for elective or planned hospitalizations, defined as hospital admissions for chemotherapy, planned surgeries or other elective procedures, chemotherapy desensitization, or bone marrow transplantation.

### Study Measures

#### Sociodemographic and Clinical Factors

We conducted an EHR review to collect demographic information (age, sex, race, education, insurance) as well as clinical factors (Charlson Comorbidity Index score,^20^ time from diagnosis of advanced cancer to index hospitalization, cancer diagnosis). For Charlson Comorbidity Index score, the patients’ malignancy was not included in the score.

#### Social Support

We used ClinicalRegex NLP software to search all clinical documentation data from the EHR for social support documentation, consistent with prior studies using NLP.^14-16^ We focused on social support documentation that occurred within 6 months of the date of index hospitalization. Our ontology for identifying social support documentation included a keyword library prioritizing sensitivity over specificity and involved 3 keyword categories: (1) social support; (2) living situation; and (3) caregivers (Supplementar Fig. S1). To identify the keywords and categories, 2 researchers (C.J., S.B.) manually reviewed 30 medical records to identify documentation of social support. The researchers identified any words or key terms that could be related to social support and organized these keywords into categories as described above. We then used the keyword library to enumerate clinical documentation about social support for each patient. We reviewed the clinical documentation enumerated by NLP and assessed social support as “limited” or “adequate” for each patient. For example, clinical documentation noting “a large support system,” or “son…living with her as well to assist in her care” resulted in enumeration of adequate social support, whereas clinical documentation noting “Family not very involved” or “limited social support” resulted in enumeration of limited social support (Supplementary Table S1). Two independent coders (C.J., S.B.) validated interrater reliability of the NLP coding method on a subset of 50 patients, achieving a Kappa of 0.90 (95% CI [CI]: 0.84-1.00). For a subset of patients (*n* = 9), we could not obtain the clinical documentation file necessary to run the NLP algorithm. For these cases, 2 coders (C.J., S.B.) used manual review of the electronic documentation to assess the extent of social support as “limited” or “adequate”. These 2 coders discussed disagreement in the manual review process with a consensus panel (C.J., S.B., A.E.J.) until reaching consensus, similar to prior studies.^21^

### Outcome Measures

We reviewed the EHR to determine date of death and date of last follow-up for all patients. We also captured information regarding unplanned readmission within 90 days of index hospitalization discharge (yes or no) and date of hospital readmission. For healthcare utilization outcomes, we excluded patients who died during the index hospitalization. We created a composite outcome of death and/or readmission within 90 days versus those alive without a readmission within 90 days to account for early mortality, as done in prior work.^18^ To further account for mortality, given that early death impacts the time at risk for readmission, we used time to first readmission within 90 days of hospital discharge as an outcome measure. This outcome was defined as the number of days from hospital discharge to first unplanned readmission within 90 days with censoring at death, as done in prior research.^18^ We determined length of stay (LOS) for the index hospitalization for each patient.

### Statistical Analysis

We used descriptive statistics to summarize patients’ sociodemographic and clinical characteristics, as well as healthcare utilization and mortality. To investigate the relationship between social support and OS, we conducted Cox proportional hazards regression analyses adjusting for the following covariates that we defined a priori based on our review of the literature and prior studies: age, sex, race, education, insurance, comorbidity score (Charlson Comorbidity Index score), time since advanced cancer diagnosis, and cancer type (gastrointestinal vs lung vs genitourinary/breast vs other).^17,18,22-28^ We did not include marital status as a covariate, given that marital status was included in the keyword library to evaluate social support. We used logistic regression models adjusting for the same covariates described above to assess the relationship between social support and the odds of death or readmission within 90 days. In addition, we used Cox proportional hazards regression models adjusting for the same covariates described above to assess the relationship between social support and time to readmission within 90 days. We used linear regression models, adjusting for the same covariates, to assess the relationship between social support and index hospitalization LOS. Because we identified differences in extent of social support among cancer types, we explored cancer type as an effect modifier of the relationship among social support with the outcomes of OS, death or readmission within 90 days, time to readmission within 90 days, and LOS. To assess this, we included an interaction term in the Cox regression analysis (cancer type × social support). We considered interaction terms with *P* < .15 to indicate potential moderation worth exploring subsequent subgroup differences, as in prior studies.^29,30^ After identifying a potential interaction, we conducted univariate analyses to examine the relationship between social support and outcomes of interest within subgroups given the limited sample size for multivariable analyses and hypothesis-generating focus of this exploratory analysis. All reported P values are 2-sided with a *P* value < .05 considered statistically significant. We performed statistical analyses using Stata version 14.2.

## Results

### Study Participants

Table 1 describes the clinical characteristics of the patients (*n* = 966) in this analysis. Patients had a median age of 65 (range: 21-92) years, and half (50.1%, 484/966) were female. The majority of patients were White (92.3%, 892/966), married (65.9%, 637/966), and educated beyond high school (68.4%, 661/966). Patients had a median Charlson Comorbidity Index score of 0 (range: 0-8). A plurality of patients had gastrointestinal cancer (34.3%), followed by lung (19.5%), and genitourinary (11.6%) cancers.

**Table 1.** Patient characteristics.

Characteristic	*N* = 966	%
Age (years)—median (range)	65 (21-92)	
Female sex	484	50.1
White race	892	92.3
Relationship status		
Single or never married	150	15.5
Married or cohabiting as if married	637	65.9
Divorced/separated	110	11.4
Widowed	69	7.1
Education		
High school or below	305	31.6
Other	661	68.4
Insurance		
Government sponsored	487	50.4
Other	479	49.6
Charlson Comorbidity Index score—median (range)	0 (0-8)	
Days from cancer diagnosis to index admission—median (range)	243 (0-5183)	
Social support		
Limited	60	6.2
Adequate	906	93.8
Cancer type		
Gastrointestinal	331	34.3
Lung	188	19.5
Genitourinary	112	11.6
Melanoma	90	9.3
Breast	75	7.8
Head and neck	54	5.6
Gynecologic	52	5.4
Sarcoma	50	5.2
Cancer of unknown primary	14	1.5

### Social Support Assessed via NLP

Overall, 6.2% (60/966) of patients had limited social support when assessed via NLP (Table 1). Among patients with gastrointestinal cancer, lung cancer, genitourinary or breast cancer, and other solid tumor cancers, the rates of limited social support assessed by NLP were 4.3% (14/331), 5.3% (10/188), 6.4% (12/187), and 9.3% (24/260), respectively.

### Survival and Healthcare Utilization

With a median follow up of 123.5 days (range: 2-2307) from date of index hospital admission to date of death or last follow up, 92.8% (896/966) of patients died. Overall, 4.4% (42/966) of patients died during the index admission. Among all patients, 42.9% (414/966) had a hospital readmission within 90 days of discharge from the index hospitalization. The median LOS for index hospitalization was 5 days (range: 1-32).

### Association Between Social Support and Overall Survival

Limited social support was not significantly associated with OS in unadjusted (HR 1.11, 95% CI: 0.84-1.46, *P* = .457) or multivariable (HR = 1.13, 95% CI: 0.86-1.49, *P* = .390) Cox regression models [Table 2]. Marital status was not significantly associated with OS (HR 1.00, 95% CI: 0.86-1.15, *P* = .987).

**Table 2.** Association of social support with overall survival.

Variable	Hazard ratio (95% CI)	SE	*P*-value
Limited social support	1.13 (0.86-1.49)	0.16	.390
Age	1.01 (1.00-1.01)	<0.01	.009
Female sex	0.89 (0.78-1.02)	0.06	.089
White race	0.97 (0.75-1.25)	0.13	.811
High school education or below	1.09 (0.94-1.26)	0.08	.238
Charlson Comorbidity Index score	1.03 (0.97-1.08)	0.28	.332
Days from advanced cancer diagnosis to admission	1.00 (1.00-1.00)	<0.01	.003
Government-sponsored insurance	0.96 (0.82-1.12)	0.08	.60
Cancer diagnosis			
Other	Ref	Ref	Ref
Gastrointestinal	1.28 (1.08-1.52)	0.11	.005
Lung	1.15 (0.94-1.41)	0.12	.178
Genitourinary/breast	1.30 (1.06-1.58)	0.13	.012

### Association Between Social Support and Healthcare Utilization

In unadjusted analyses, limited social support was not associated with death or readmission within 90 days of discharge from the index hospitalization (OR = 1.20, 95% CI: 0.68-2.11, *P* = .538), time to readmission within 90 days (HR = 1.00, 95% CI: 0.67-1.47, *P* = .980), or index hospitalization LOS (β= −0.06, 95% CI: −1.31 to 1.19, *P* = .924). In multivariable models, limited social support was not associated with these healthcare utilization outcomes (Tables 3 and 4).

**Table 3.** Association of social support with death or readmission within 90 days.

Variable	Odds ratio (95% CI)	SE	*P*-value
Limited social support	1.18 (0.66-2.12)	0.35	.578
Age	1.00 (0.99-1.02)	<0.01	.516
Female sex	0.89 (0.68-1.18)	0.13	.430
White race	0.97 (0.75-1.25)	0.13	.811
High school education or below	1.11 (0.66-1.86)	0.29	.693
Charlson Comorbidity Index score	1.20 (1.06-1.35)	0.08	.005
Days from advanced cancer diagnosis to admission	1.00 (1.00-1.00)	<0.01	.001
Government-sponsored insurance	0.73 (0.52-1.01)	0.12	.058
Cancer diagnosis			
Other	Ref	Ref	Ref
Gastrointestinal	1.33 (0.94-1.89)	0.24	.111
Lung	1.02 (0.68-1.54)	0.21	.906
Genitourinary/breast	1.40 (0.92-2.12)	0.30	.114

**Table 4.** Association of social support with time to readmission within 90 days.

Variable	HR (95% CI)	SE	*P*-value
Limited social support	0.92 (0.62-1.38)	0.19	.698
Age	0.99 (0.98-1.00)	<0.01	.184
Female sex	0.89 (0.68-1.18)	0.13	.430
White race	1.05 (0.73-1.51)	0.20	.806
High school education or below	1.10 (0.89-1.35)	0.12	.385
Charlson Comorbidity Index score	1.11 (1.03-1.20)	0.04	.005
Days from advanced cancer diagnosis to admission	1.00 (1.00-1.00)	<0.01	.004
Government-sponsored insurance	0.95 (0.75-1.20)	0.11	.673
Cancer diagnosis			
Other	Ref	Ref	Ref
Gastrointestinal	1.14 (0.89-1.46)	0.14	.298
Lung	0.88 (0.65-1.20)	0.14	.419
Genitourinary/breast	1.26 (0.94-1.68)	0.19	.129

### Limited Social Support and Outcomes Among Cancer Types

We identified a potential interaction suggesting cancer type as an effect modifier of the relationship between social support and overall survival (interaction term *P* = .053), but not with death or readmission within 90 days of discharge (interaction term *P* = .613), time to hospital readmission (interaction term *P* = .557), or hospital LOS (interaction term *P* = .299). In an unadjusted Cox regression model among patients with gastrointestinal cancers, limited social support was associated with significantly worse OS (HR = 2.10, 95% CI: 1.22-3.62, *P* = .008), but not among patients with other cancers (HR = 1.00, 95% CI: 0.73-1.37, *P* = .991). Marital status was not significantly associated with OS among patients with gastrointestinal cancers (HR = 1.10, 95% CI: 0.87-1.39, *P* = .442).

## Discussion

In this study, we found that NLP could help to evaluate the extent of social support in adults with advanced cancer. We did not find associations of limited social support with OS, death or readmission within 90 days of discharge, time to hospital readmission, or hospital LOS. However, we did identify a differential impact of social support on survival among gastrointestinal cancers versus other cancer types. These findings underscore the need for additional larger studies evaluating the association of social support with clinical outcomes in specific cancer types.

Our findings highlight the use of NLP as a method for extracting social support documentation from the EHR and underscore the use of NLP as a novel tool in oncology. Our analysis applied NLP to assess the extent of social support in more than 900 patients and demonstrated excellent inter-rater reliability, illustrating how NLP can successfully harness data to evaluate social support in large cohorts of patients with cancer. Prior work has assessed social support in solid tumors predominately using marital status or patient self-report.^25^ In contrast, NLP provides the capacity to measure social support across multiple dimensions and allows for the rapid analysis of vast unstructured EHR text information.^14^ Social support represents a critical aspect of patients’ health-related quality of life and mood symptoms in oncology.^31,32^ Therefore, by helping to determine those in need of additional support, NLP could be instrumental in improving outcomes for individuals with cancer. Collectively, our findings suggest that health systems could potentially integrate NLP into the EHR to further examine social support in patients with cancer and other significant medical comorbidities as a mechanism for identifying those in need of additional support services. Further validation of a social support keyword library could allow for identification of those in need of social support interventions across EHR systems with unstructured text information and permit the testing and evaluation of targeted social support interventions.

Contrary to our hypothesis, we did not identify a statistically significant association between limited social support and OS in hospitalized adults with advanced cancer. Prior research has shown mixed results with respect to social support and survival among different cancer types, with one study reporting an association between social isolation and OS in patients with breast cancer,^11^ whereas another in patients with non–small cell lung cancer reported no association between marital status and survival.^12^ Additionally, a meta-analysis found a relationship among perceived social support, social network size, and marital status with OS in patients with cancer, yet with notable differences among cancer types.^25^ Our work differs from prior investigations by focusing on patients with advanced cancer and through the use of NLP to assess the extent of social support.

Although we found no significant associations between social support and survival when assessing the overall solid tumor population, we identified differential associations based upon cancer type. In the subgroup of patients with gastrointestinal malignancies, we found that limited social support was associated with worse OS. In contrast, we did not identify significant associations among the other cancer types. Notably, patients with advanced gastrointestinal malignancies experience high symptom burden, intensive treatments, and frequent hospitalizations.^33-36^ Consequently, social support may be particularly salient for this population. Furthermore, previous work examining social support via NLP in patients with aggressive hematologic malignancies demonstrated an association between limited social support with worse overall survival.^5^ Social support may be especially crucial in patients with aggressive hematologic malignancies, as these patients often endure immense physical and psychological symptoms, receive intensive therapies with significant toxicities and adverse effects, and have unique illness trajectories.^37^ These findings support that cancer type is a critical factor in the relationship between social support and survival. Future work with more robust sample sizes in various cancers should further explore the relationships among tumor type, social support, and health outcomes.

We also found no significant associations between social support and healthcare utilization, including with death or readmission within 90 days of discharge, time to hospital readmission, or LOS in this patient population. Prior work including older adults in the general medicine population has demonstrated that social support correlates with early hospital readmissions and longer hospital LOS.^38^ Patients with advanced cancer have high symptom burden and rates of healthcare utilization,^17,21^ and thus we hypothesized that limited social support would correlate with significantly higher rates of death or readmission within 90 days of discharge, decreased time to hospital readmission, and increased LOS in this population. The cancer types in our study were heterogenous with differential risk for healthcare utilization.^39^ The heterogeneity of the study patient population, particularly with respect to cancer types, as well as the low rates of limited social support among patients in our study may have resulted in lack of adequate statistical power to fully explore the relationship between social support and healthcare utilization, especially across different cancer types. Future studies further examining the impact of social support on healthcare utilization in these distinct populations are warranted.

Our study has several limitations to consider. First, this secondary analysis included patients seen at a single academic tertiary care center where the majority of patients are White and highly educated. Thus, the study population had limited diversity and relatively high social support, and our findings may not generalize to other populations. Second, our NLP model only detects phrases in notes that align with previously specified keywords, which can result in missed social support documentation. Our NLP algorithm used a broad range of keywords to ensure that we captured as much relevant documentation as possible. Third, our searches were limited to patient information accessible in our EHR, and therefore we lack data about admissions and healthcare utilization at outside institutions. Fourth, the extent of EHR documentation regarding social support could possibly reflect the degree of support provided and documented by healthcare clinicians, and we lack information regarding the type of clinician (ie, physician, psychologist, registered nurse, social worker) who entered the social support information. In addition, NLP analysis depends upon clinician documentation of information pertinent to social support. Lastly, our sample size limited the number of predictors we could include in our multivariable analyses, and thus we had insufficient power to evaluate associations of social support with OS among individual cancer types in multivariable regression models.

## Conclusion

We used NLP to assess the extent of social support in a large cohort of patients with advanced cancer. We did not find significant associations of limited social support with clinical outcomes, including OS, likelihood of death or readmission within 90 days of discharge, time to readmission, or LOS. However, we detected differential associations among cancer types and identified an association of limited social support with survival in those with gastrointestinal malignancies. This study supports the use of NLP as a tool to extract and assess social support in patients with cancer. Future work is needed to further explore the integration of NLP into the EHR to assess complex health constructs. Larger scale studies should further examine the impact of social support on outcomes in patients with advanced cancer, especially those with gastrointestinal malignancies.

## Supplementary Material

oyac238_suppl_Supplementary_Figure_S1

oyac238_suppl_Supplementary_Table_S1

## Data Availability

The data underlying this article will be shared on reasonable request to the corresponding author.
