# Supplementary material for: The Use of Natural Language Processing to Assess Social Support in Patients With Advanced Cancer
Source: Oncologist. 2022 Nov 25;28(2):165–71. doi: 10.1093/oncolo/oyac238 (PMC9907037; doi:10.1093/oncolo/oyac238)
Supplement: oyac238_suppl_Supplementary_Figure_S1 [file oyac238_suppl_supplementary_figure_s1.docx]

**Supplemental Figure 1.** Natural Language Processing Keyword Library

| Domain | Keywords |
| --- | --- |
| Social Support | involved/supportive, social support, supportive marriage, limited support, minimal support, supportive wife, supportive husband, supportive partner, supportive partnership, supportive significant other, supportive family, person who will assist patient, supportive caregiver, supportive friend, supportive friends, supportive brother, supportive sister, supportive children, supportive child, supportive son, supportive daughter, supportive sons, supportive daughters, supportive spouse, supportive parent, supportive father, supportive dad, supportive mother, supportive mom, son involved, daughter involved, sons involved, daughters involved, child involved, supportive girlfriend, supportive boyfriend, supportive fiancé, many friends, good support, close with her, close with his, close with their, support system, identified social support, well supported, well-supported, great support, poor support, no support, family support, practical support, supportive parents, children involved, wife involved, husband involved, community support, supportive community, engaged with family, family conflict, been fighting with, marriage conflict, poor relationship, limited contact with, not speaking with, not speaking to, relationship conflict, spouse involved, fiancé involved, girlfriend involved, boyfriend involved, parents involved, parent involved, father involved, mother involved, dad involved, mom involved, supportive sibling, sibling involved, supportive aunt, supportive uncle, supportive niece, supportive nephew, aunt involved, uncle involved, niece involved, nephew involved, supportive grandparents, grandparents involved, supportive grandparent, grandparent involved, supportive grandmother, grandmother involved, supportive grandfather, grandfather involved |
