# Supplementary material for: The Use of Natural Language Processing to Assess Social Support in Patients With Advanced Cancer
Source: Oncologist. 2022 Nov 25;28(2):165–71. doi: 10.1093/oncolo/oyac238 (PMC9907037; doi:10.1093/oncolo/oyac238)
Supplement: oyac238_suppl_Supplementary_Table_S1 [file oyac238_suppl_supplementary_table_s1.docx]

**Supplemental Table 1.** Examples of Social Support Assessment via Natural Language Processing

| Assessment | Keyword | Phrase in Clinical Documentation |
| --- | --- | --- |
| Adequate Social Support | ‘Support system’ | “Patient also has a large support system of friends and extended family.” |
| Adequate Social Support | ‘Lives with’ | “Lives with her husband, son says he has been living with her as well to assist in her care. She will not be home alone at any time.” |
| Limited Social Support | ‘Divorced’; ‘lives alone’; ‘lives with’ | “Pt divorced and lives alone in apt, has 24yo son who lives with ex-wife…Family not very involved.” |
| Limited Social Support | ‘living alone’; ‘social support’; ‘lives alone’ | “Pt previously living alone…limited social support and home stairs are potential barriers to her rehab potential…Pt lives alone” |
